# Decoy receptor 3 (DcR3) overexpression predicts the prognosis and pN2 in pancreatic head carcinoma

**DOI:** 10.1186/1477-7819-12-52

**Published:** 2014-03-05

**Authors:** Jian Zhou, Shiduo Song, Dechun Li, Songbing He, Bing Zhang, Zhenxin Wang, Xinguo Zhu

**Affiliations:** 1 Department of General Surgery, The First Affiliated Hospital of Soochow University, 188 Shizi Street, Suzhou 215006, China; 2 Department of Oncology, The First Affiliated Hospital of Soochow University, 188 Shizi Street, Suzhou 215006, China; 3 Department of Nuclear Medicine, The First Affiliated Hospital of Soochow University, 188 Shizi Street, Suzhou 215006, China

**Keywords:** DcR3, Lymphadenectomy, Pancreatic head carcinoma, Prognosis

## Abstract

**Background:**

This study was carried out to examine decoy receptor 3 (DcR3) expression and investigate its clinical and prognostic significance in patients with pancreatic head carcinoma.

**Methods:**

Tissue samples were obtained from 50 patients with pancreatic head carcinoma. DcR3 protein expression in tissues and sera was assessed by immunohistochemistry and ELISA. Correlations between DcR3 and clinicopathologic features and prognoses were analyzed statistically.

**Results:**

Serum DcR3 levels were significantly elevated in patients with pancreatic head carcinoma compared with patients with cystadenoma and healthy individuals (*P <* 0.01 and *P <* 0.01, respectively). DcR3 overexpression correlated with lymph node metastases and TNM stages (*P <* 0.05 and *P <* 0.05, respectively). Median overall survival for the high DcR3 group was 16.3 months, compared to 21.6 months for the low DcR3 group (*P <* 0.05). In the low DcR3 group, no significant difference was found in the overall survival between patients who underwent standard pancreatoduodenectomy (SPD) and those who had radical pancreatoduodenectomy (RPD) (*P >* 0.05). In the high DcR3 group, the median overall survival rates were 16.8 months in the RPD group and 13.5 months in the SPD group (*P <* 0.05).

**Conclusions:**

We found that DcR3 was overexpressed in pancreatic head carcinoma. The patients with high DcR3 levels had higher pN2 stages than those with low DcR3 levels. Detecting serum DcR3 level preoperatively might be an additional approach for evaluating pN2 stage and guiding the range of lymphadenectomy.

## Background

Pancreatic carcinoma is one of the most lethal and aggressive of all malignancies. It is the fourth leading cause of cancer-related deaths in the Western world and leads to an estimated 277,000 deaths per year worldwide [1]. The median overall survival of patients with pancreatic carcinoma is less than 6 months, and the 5-year survival rate is dismal at 2% to 6% [2]. Despite advances in chemotherapy and radiotherapy over the past few decades, the overall prognosis for patients with pancreatic carcinoma has remained essentially unchanged [3]. Surgical resection is the only curative therapy if the tumor is limited and without vascular encasement or distant metastases.

About 65% of pancreatic carcinomas are in the head of the gland, approximately 25% are in the body and tail and the remainder are multifocal. Pancreatoduodenectomy (PD) is considered the operation of choice for pancreatic head carcinoma. According to the extent of lymphadenectomy, three different procedures have been identified [4]: standard pancreatoduodenectomy (SPD), radical pancreatoduodenectomy (RPD) and extended radical pancreatoduodenectomy (ERPD). In theory, there can be no doubt that RPD increases the R0 resection rate compared with SPD. However, its influence on short-term postoperative complications and mortality, as well as its benefit in terms of long-term survival, is a matter of debate [5-7]. Therefore, an ideal surgical procedure for pancreatic head carcinoma is to perform adapted surgery for each patient on the basis of accurate preoperative evaluation of lymph node (LN) stage to determine the range of lymphadenectomy.

Helical computed tomography (CT), endoscopic ultrasonography (EUS) and magnetic resonance imaging play a key role in detecting small lesions and assessing local invasion and distant metastases, and they provide about 80% accuracy regarding resectability and 90% accuracy for unresectability [8,9]. All of these imaging methods are limited in their ability to detect metastatic LNs in pancreatic carcinoma, however, especially those less than 10 mm in diameter [10]. Because of these imaging procedure limitations, some molecules have been focused on for the evaluation of LN status. Decoy receptor 3 (DcR3), a newly discovered member of the tumor necrosis factor receptor (TNFR) superfamily, is regarded as a secreted molecule because it lacks a transmembrane sequence [11]. There is strong evidence that DcR3 is overexpressed in malignant tumors of the stomach, lung, liver and ovary [12-15]. We previously reported that DcR3 mRNA was closely associated with LN metastasis and TNM stage in pancreatic carcinoma [16]. Moreover, a report by Wu *et al*. demonstrated that serum DcR3 levels were related to pathological LN (pN) staging and distant metastases in gastric carcinoma [17].

We speculated that DcR3 might be a potential marker for predicting LN status preoperatively to guide the range of lymphadenectomy in pancreatic head carcinoma. In this study, we investigated DcR3 expression in 50 patients with pancreatic head carcinoma and analyzed the relationship between serum DcR3 level and LN stage. On the basis of our results, we suggest that DcR3 plays a substantial role in determining the surgical approach to be used in lymphadenectomy.

## Methods

### Ethical approval

The project was carried out according to the Declaration of Helsinki and was approved by the local ethics committee.

### Clinical data

Fifty patients underwent PD for pancreatic head carcinoma at the First Affiliated Hospital of Soochow University between November 2007 and February 2011. All clinical pathologic features were prospectively documented, including age, sex, tumor size and LN stage. Grades of differentiation and TNM stages were assigned according to the World Health Organization [18] and Union for International Cancer Control [19] classification systems. The Japanese Pancreas Society rules were chosen to define the LN stations and the extent of LN dissection precisely [20]. N1 stations included 12 (b1, b2 and c), 13 (a and b), 14 (a and b), (a and b) and 8a. N2 stations included all of 8, 12, 14 and 9, as well as 16 (a2 and b1). According to the extent of lymphadenectomy, SPD comprises a pancreatoduodenectomy plus resection of N1 stations, and RPD comprises cleaning N2 stations together with those removed during SPD. SPD or RPD was performed if the tumor was resectable. Tumorous tissues and adjacent nontumorous tissues were frozen in liquid nitrogen immediately after surgical removal and stored at -80°C. Serum samples were obtained from tumor patients for enzyme-linked immunosorbent assays (ELISAs) before surgery. Sera of healthy individuals were used as controls. The patients did not receive any chemotherapy, radiotherapy or immunotherapy.

### Immunohistochemistry

Tissues were fixed with formalin and embedded in paraffin. Sections 4 μm thick were mounted on glass slides pretreated with 0.1% poly-L-lysine and incubated in anti-DcR3 antibody (Abcam, Cambridge, UK) at 10 μg/ml overnight at 4°C. Immunoreactivity was detected by avidin-biotin complex with 3,3′-diaminobenzidine substrate. Sections were evaluated by light microscopy for staining intensity and quantity. The staining intensity was scored as follows: 0 = negative, 1 = low, 2 = medium and 3 = high. The quantity was subdivided as follows: 0 = ≤5%, 1 = 6% to 25%, 2 = 26% to 50%, 3 = 51% to 75% and 4 = >75%. The final score was calculated by multiplication of the intensity score by the quantity score. Scores ≥6 were considered to indicate positive expression.

### Enzyme-linked immunosorbent assay

The level of DcR3 in human serum was measured by using a commercially available sandwich ELISA kit (R&D Systems, Minneapolis, MN, USA). Procedures were conducted as suggested by the manufacturers.

### Statistical analysis

SPSS version 16.0 software (IBM SPSS, Chicago, IL, USA) was used for statistical analysis. Clinical variables were compared between groups using the Mann-Whitney *U* test or the Kruskal-Wallis H test for continuous variables and Fisher’s exact test for categorical variables. Patients were divided into two groups based on serum DcR3 levels: those at or above the median (high DcR3) and those below the median (low DcR3). Overall survival was calculated by the Kaplan-Meier method, and the significance of differences was determined by applying the logrank test. Multivariate analysis was performed using the Cox proportional hazards regression model. *P <* 0.05 was considered statistically significant.

## Results

### Overexpression of DcR3 in pancreatic head carcinoma

**Figure 1:**
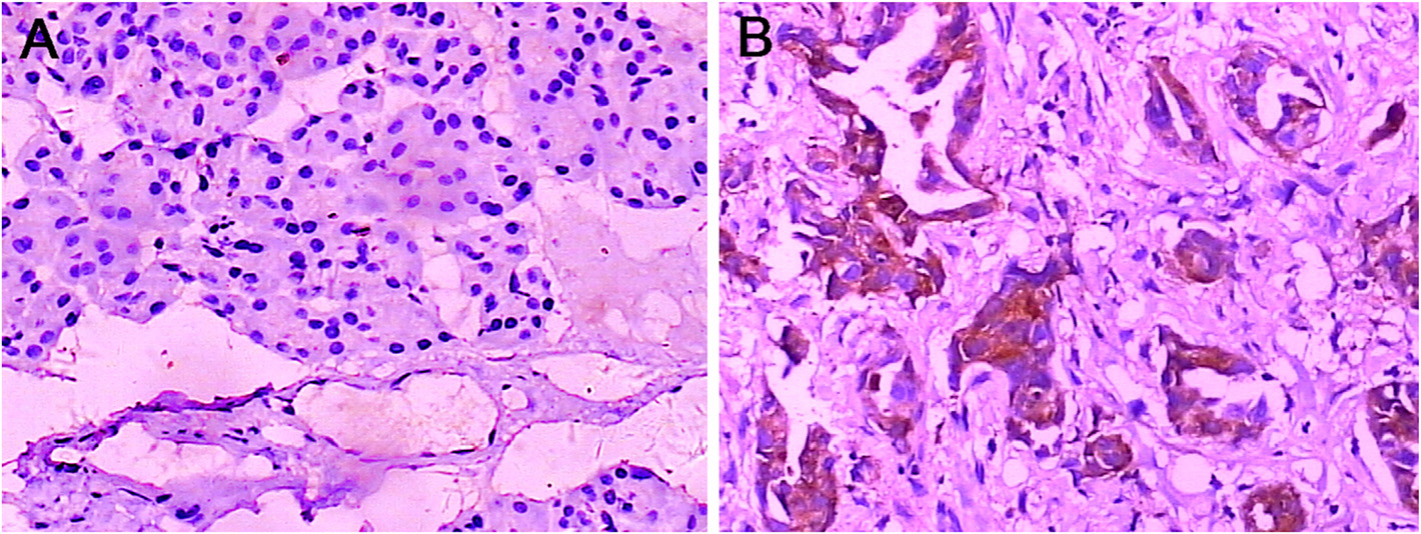
**Immunohistochemical staining of DcR3 protein. (A)** Image showing high expression of decoy receptor 3 (DcR3) protein in pancreatic carcinoma tissues. **(B)** Image showing low expression of DcR3 in pancreatic nontumorous tissues. Original magnification, ×200.

**Figure 2:**
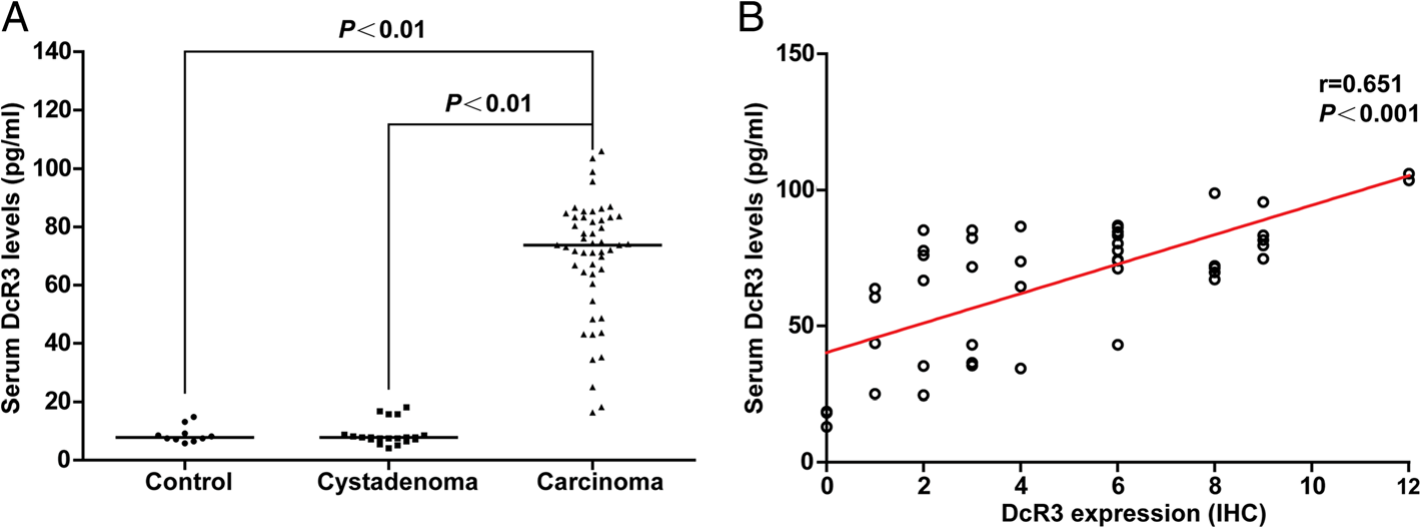
**Expression levels of DcR3 in pancreatic carcinoma. (A)** The serum levels of decoy receptor 3 (DcR3) protein were detected by enzyme-linked immunosorbent assay (ELISA). **(B)** Comparison of DcR3 serum concentrations measured by ELISA and DcR3 expression by immunohistochemistry (IHC).

In 50 patients who had recently undergone surgery for pancreatic head carcinoma, paired samples of tumorous and nontumorous tissues were studied by immunohistochemistry (IHC). The subcellular expression pattern of DcR3 was diffusely cytoplasmic mainly in pancreatic carcinoma tissue, and the nontumorous tissues were negative or weakly positive for DcR3 (Figures 1A and 1B). The positive rate of DcR3 in tumor tissues was 78% (39 of 50) and that in nontumorous tissues was 24% (12 of 50), a significant difference (*P <* 0.01). Because DcR3 lacks a transmembrane sequence and is a soluble protein, we performed ELISA to determine the serum levels of DcR3. As shown in Figure 2A, serum DcR3 levels were significantly elevated in patients with pancreatic head carcinoma compared to cystadenoma patients or healthy individuals (*P <* 0.01 and *P <* 0.01, respectively). Moreover, the serum levels of DcR3 were associated with DcR3 expression as determined by IHC in the tumor tissues (*P <* 0.01) (Figure 2B).

### Serum DcR3 levels in relation to lymph node metastasis and TNM stages

The expression of DcR3 was analyzed in comparison with clinical pathological factors. As shown in Table 1, there were no significant differences with regard to age, sex, tumor grade and tumor size when the group was divided at the median serum DcR3 level. However, the difference of LNs between the low DcR3 and high DcR3 groups was significant (*P <* 0.05). Of patients with high DcR3 levels, 88% (22 of 25) fell into the LN metastasis group compared to only 32% (8 of 25) in the low DcR3 group (*P <* 0.05). With respect to clinical stages, DcR3 expression also had a noteworthy association (*P <* 0.05). Thus, the serum level of DcR3 was related to LN metastases and TNM stages, but was not correlated with any other factors at a statistically significant level.

**Table 1:** Correlation of DcR3 expression with clinical pathological factors

**Characteristics**	**Low DcR3 group (*n*** **= 25)**	**High DcR3 group (*n*** **= 25)**	***P***-**value**
Sex			0.951
Female	11	9	
Male	14	16	
Age (years)	58 ± 10	60 ± 12	0.426
Tumor size			0.081
≤2 cm	15	8	
>2 cm	10	17	
Tumor grades			0.389
G1	10	9	
G2/3	15	16	
Lymph node metastases			0.001
Negative	17	3	
Positive	8	22	
TNM stage			0.007
I	15	4	
II	10	21	

### Prognostic significance of DcR3 overexpression for survival

**Figure 3:**
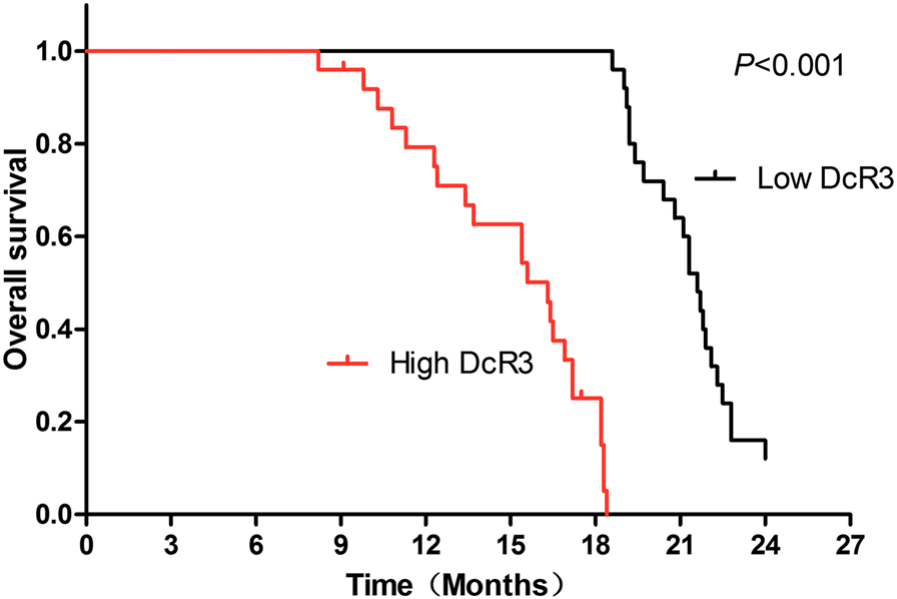
**Univariate analyses of overall survival in patients separated into high DcR3 and low DcR3 groups.** Kaplan-Meier survival curves are shown. The logrank test was used to calculate *P*-values. Compared to low decoy receptor 3 (DcR3) protein expression (below the median; n = 25), high expression of DcR3 (at or above the median; *n* = 25) was correlated with shorter overall survival.

Univariate survival analysis showed that DcR3 overexpression was associated with a significantly shortened duration of overall survival. As shown in Figure 3, median overall survival for the high DcR3 group was 16.3 months compared to 21.6 months for the low DcR3 group (*P <* 0.05). Multivariate Cox proportional hazards analysis was performed to investigate the impact of DcR3 expression on the cancer-specific survival. As shown in Table 2, tumor size, LN metastasis, TNM stage and DcR3 expression were independent prognostic factors for survival.

**Table 2:** **Multivariate Cox proportional hazards analysis in pancreatic head carcinoma**^**a**^

**Prognostic factors**	**Hazard ratio**	**95% CI**	***P*-value**^**b**^
Sex (female or male)	1.46	0.82 to 2.96	0.31
Age (<60 or ≥60 years)	1.54	0.68 to 3.26	0.29
Tumor size (≤2 cm or >2 cm)	1.16	0.63 to 2.08	0.03
Tumor grade (G1 or G2/3)	1.62	0.86 to 3.28	0.13
Lymph node metastases (negative or positive)	1.18	0.62 to 2.24	0.03
High vs. low DcR3			
TNM stage (I or II)	0.85	0.44 to 1.76	0.02
DcR3 expression (high or low)	1.18	0.88 to 3.36	0.04

^a^DcR3, Decoy receptor 3 protein. ^b^*P* < 0.05 was statistically significant.

### Correlation between DcR3 expression and pathological lymph node stages in pancreatic head carcinoma

**Figure 4:**
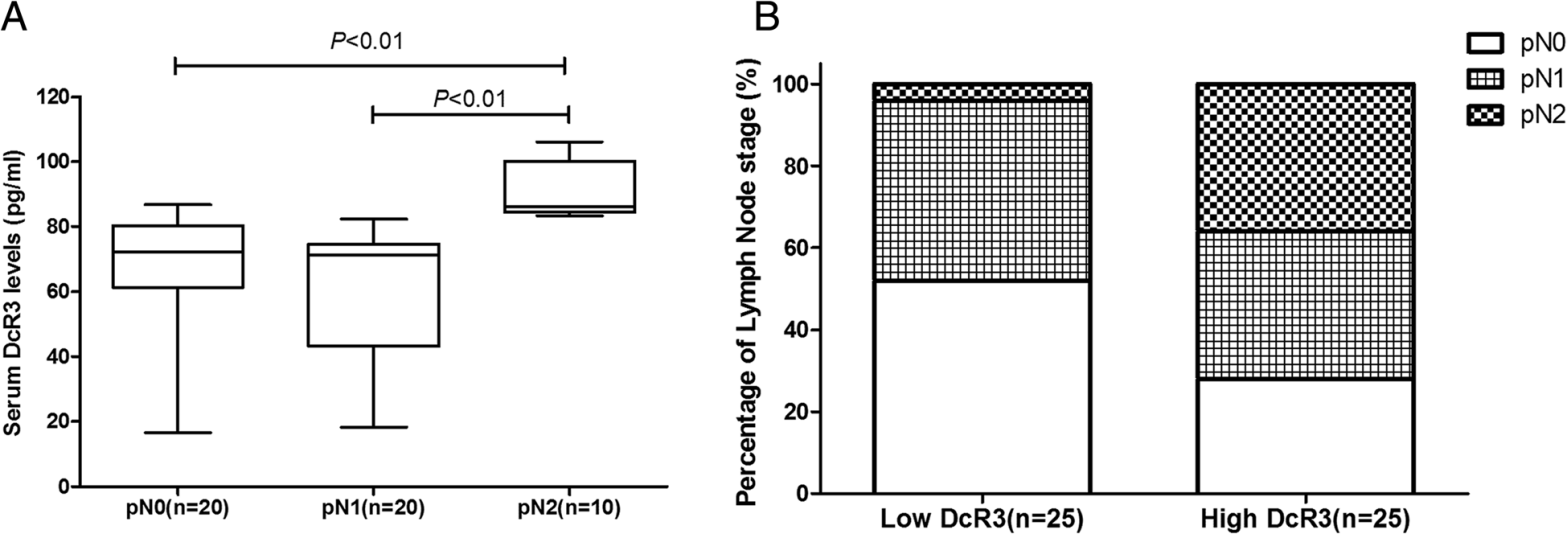
**Correlation between DcR3 expression levels and pN stages. (A)** Comparison of decoy receptor 3 (DcR3) protein expression levels between patients with pathological lymph node stage 0 (pN0) disease (*n* = 20), pN1 disease (*n* = 20) and pN2 disease (*n* = 10). **(B)** Distribution of pN stages according to DcR3 expression.

As shown in Figure 4A, the median levels of DcR3 expression were 72.05 pg/ml for pathological lymph node stage 0 (pN0), 71.23 pg/ml for pN1 and 86.13 pg/ml for pN2. Patients with pN2 tumors had much higher levels of DcR3 expression than those with pN0 or pN1 tumors (*P <* 0.01 for both comparisons). A strong correlation was noted between DcR3 expression and pN stage (Figure 4B). In the low DcR3 group, 4% (1 of 25) were staged as pN2, and the remaining 96% (24 of 25) had pN0 or pN1 tumors. In the high DcR3 group, 36% (9 of 25) had pN2 tumors. The results indicate that high DcR3 expression was more frequently involved in pN2 stage tumors than low DcR3 expression (36% vs. 4%; *P <* 0.01).

### Effect on survival according to levels of DcR3 expression and surgical procedure

**Figure 5:**
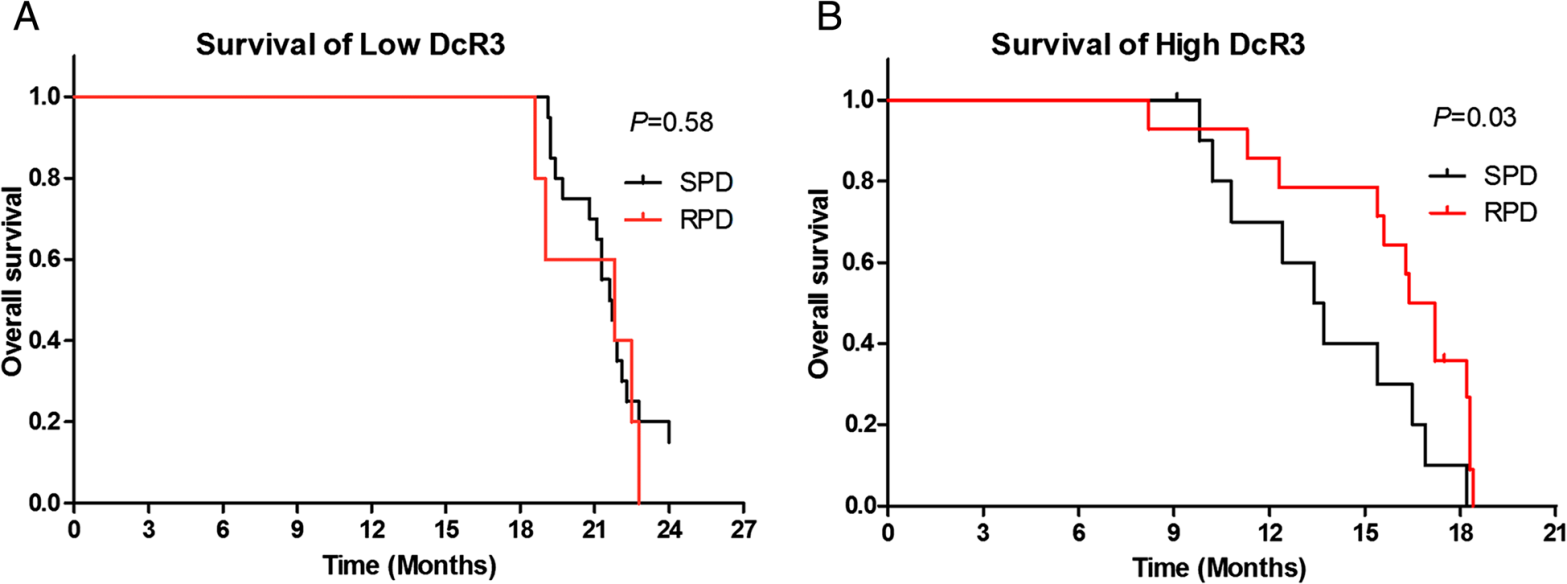
**Kaplan-Meier curves for overall survival in patients with different DcR3 levels who underwent standard pancreatoduodenectomy or radical pancreatoduodenectomy. (A)** Patients with low decoy receptor 3 (DcR3) protein levels (*n* = 25). **(B)** Patients with high DcR3 levels (*n* = 25). RPD, radical pancreatoduodenectomy; SPD, Standard pancreatoduodenectomy.

The prognostic significance of the surgical procedure performed in relation to patient survival was evaluated at different DcR3 levels by univariate analysis. As shown in Figure 5A, in the low DcR3 group, no significant difference in overall survival was found between patients who underwent SPD compared with those who underwent RPD (*P >* 0.05). In the high DcR3 group, the median overall survival durations were 16.8 months for RPD patients and 13.5 months for SPD patients (*P <* 0.05) (Figure 5B). These data indicate that the patients with high DcR3 expression benefited from RPD on the basis of overall survival, despite the fact that the extent of lymphadenectomy was expanded.

## Discussion

In this study, we demonstrated that DcR3 was overexpressed in pancreatic head carcinoma. Our data suggest that serum DcR3 protein levels of patients assessed by ELISA corresponded well with immunohistochemical detection of DcR3 expression in tissues. Wu *et al*. reported that 55% of patients with liver, gastric and colon cancer were serum DcR3-positive [19]. Therefore, detection of DcR3 in serum offers an efficacious way to obtain tumor diagnostic and prognostic information.

DcR3, also known as TR6 or M68, is a new number of the TNFR superfamily that maps to chromosome position 20q13 [21]. It shares sequence homology with osteoprotegerin (31%) and TNFR2 and relatively less homology with the Fas ligand (FasL). It has been reported that DcR3 has three ligands: FasL, TNF-like molecule 1A (TL1A) and LIGHT (lymphotoxin-like, exhibits inducible expression, and competes with herpes simplex virus glycoprotein D for binding to herpes virus entry mediator, a receptor expressed by T lymphocytes). DcR3 apparently could not only block apoptosis of Fas, TL1A and LIGHT by inhibiting the FasL–Fas, TL1A–death receptor 3 (DR3) and LIGHT–lymphotoxin β receptor interactions [22-24] but also suppress immune surveillance by blocking T-cell costimulation mediated by TL1A and LIGHT [25,26]. Our previous study indicated that silencing DcR3 by lentivirus-mediated DcR3 RNAi enhances the effect of FasL and inhibits tumor growth *in vitro* and *in vivo*[27]. Moreover, DcR3 reportedly modulates the function of dendritic cells and deviates T-cell responses toward the Th2 phenotype [28,29].

According to the results of our present study, serum DcR3 levels in pancreatic head carcinoma were significantly associated with LN metastasis and TNM stage, but did not correlate with any other factors, such as age, sex, tumor grade and tumor size. Furthermore, serum levels of DcR3 in the patients with pN2 tumors were higher than serum levels in those with pN0 or pN1 tumors, and high DcR3 expression was more frequently involved than low DcR3 expression in pN2 stage tumors. However, the exact mechanism for more pN2 coupled with DcR3 overexpression is poorly understood. In addition, we have shown in this study that DcR3 overexpression was associated with significantly shorter overall survival compared with patients whose serum DcR3 levels were low. In multivariate analysis including other prognostic factors (such as tumor size, LN metastasis and TNM stage), DcR3 expression was prognostic for cancer-specific survival.

The correlation between serum levels of DcR3 and LN stages has especially useful clinical applications. The range of lymphadenectomy in PD operations depends on preoperative identification of LN status. Although a more extensive lymphadenectomy clearly increases the likelihood of complete (R0) resection, which is beneficial for the patient’s overall survival, it is also informative regarding increased postoperative complications and mortality. Surgeons need to maintain a balance between performing an unnecessarily extensive lymphadenectomy and leaving uncertainty about whether metastatic nodes can be removed. As serum DcR3 was correlated with pN2 stage, detection of DcR3 expression before lymphadenectomy might be a useful method of accurately evaluating LN stages in pancreatic carcinoma, in addition to CT and EUS. Knowledge of high DcR3 levels allows surgeons to make a decision whether to perform SPD or RPD, with the latter more likely to achieve R0 resection for pN2-positive patients. Our data indicate that there was no significant difference in overall survival between the SPD and RPD groups with low DcR3 levels. In patients with high DcR3 levels, the overall survival for the RPD group was longer than that for the SPD group, which indicates that SPD might be sufficient for patients with low DcR3 expression. In contrast, with regard to overall survival, of patients with high DcR3 levels benefited from RPD despite the expansion of the extent of lymphadenectomy.

## Conclusions

The results of this study indicate that DcR3 was overexpressed in pancreatic head carcinoma. The patients with high DcR3 levels presented higher pN2 stages than those with low DcR3 levels. Detecting serum DcR3 levels before surgery might be an additional approach to be used in evaluating pN2 stage and guiding the range of lymphadenectomy.

## Competing interests

The authors declare that they have no competing interests.

## Authors’ contributions

JZ, SS, DL and XZ conceived the study and participated in its design, data extraction, manuscript drafting and editing. SH carried out immunohistochemistry. ZW carried out ELISAs. BZ performed the statistical analysis. All authors read and approved the final manuscript.
